# Multiple malignant tumors in a patient with familial chordoma, a case report

**DOI:** 10.1186/s12920-021-01064-0

**Published:** 2021-08-31

**Authors:** Nuttavut Sumransub, Paari Murugan, Shelly Marette, Denis R. Clohisy, Keith M. Skubitz

**Affiliations:** 1 Department of Medicine, University of Minnesota, 420 Delaware St SE, Minneapolis, MN 55455 USA; 2 Department of Laboratory Medicine and Pathology, University of Minnesota, 420 Delaware St SE, Minneapolis, MN 55455 USA; 3 The Masonic Cancer Center, 425 E River Pkwy, Minneapolis, MN 55455 USA; 4 Department of Radiology, 420 Delaware St SE, Minneapolis, MN 55455 USA; 5 Department of Orthopaedic Surgery, 2450 Riverside Ave Suite R200, Minneapolis, MN 55454 USA; 6 Department of Hematology, Oncology, and Transplantation, Department of Medicine, University of Minnesota, 420 Delaware St. SE MMC 480, Minneapolis, MN 55455 USA

**Keywords:** Familial chordoma, *TBXT* gene, Brachyury, Undifferentiated pleomorphic sarcoma, Case report

## Abstract

**Background:**

Chordoma is a rare bone tumor that is typically resistant to chemotherapy and is associated with genetic abnormalities of the T-box transcription factor T (*TBXT*) gene, which encodes the transcription factor brachyury. Brachyury is felt to be a major contributor to the development of chordomas.

**Case presentation:**

We describe a 67-year-old woman who developed an undifferentiated pleomorphic sarcoma in her thigh. Despite treatment with standard chemotherapy regimens, she had a rapidly progressive course of disease with pulmonary metastases and passed away 8 months from diagnosis with pulmonary complications. Her medical history was remarkable in that she had a spheno-occipital chordoma at age 39 and later developed multiple other tumors throughout her life including Hodgkin lymphoma and squamous cell carcinoma and basal cell carcinoma of the skin. She had a family history of chordoma and her family underwent extensive genetic study in the past and were found to have a duplication of the *TBXT* gene.

**Conclusions:**

Brachyury has been found to associate with tumor progression, treatment resistance, and metastasis in various epithelial cancers, and it might play roles in tumorigenesis and aggressiveness in this patient with multiple rare tumors and germ line duplication of the *TBXT* gene. Targeting this molecule may be useful for some malignancies.

**Supplementary Information:**

The online version contains supplementary material available at 10.1186/s12920-021-01064-0.

## Background

Chordoma is a rare bone sarcoma with an incidence rate below 0.1 per 100,000 [1]. It is derived from remnants of the notochord, an embryonic structure that is required for the induction of the neural plate in the embryonic disk. Chordoma typically occurs in the skull base, mobile spine, and sacrum. Although distant metastasis may occur, chordomas usually behave as low-grade neoplasms with a locally aggressive growth pattern and high local recurrence rates. Surgery and radiotherapy are the mainstays of treatment, but many patients develop tumor recurrence or complications from treatment. These tumors typically are resistant to traditional chemotherapy and no standard treatment has been approved [2, 3].

While most cases of chordoma are sporadic, reports of two or more close relatives with chordoma suggest a genetic predisposition for this disease. Probable autosomal dominant inheritance in familial chordoma was first reported by Stepanek et al. [4]. A series of subsequent studies demonstrated duplication of the *TBXT* gene, a member of the T-box proteins encoding brachyury, that is felt to be a major susceptibility mechanism for the development of chordoma in several families [5, 6]. Brachyury is a transcription factor within the T-box family of genes that is expressed in the nuclei of notochord cells and is essential for notochord development [7]. Knocking down brachyury in a chordoma cell line resulted in decreased proliferation and cell senescence [8]. Brachyury is considered a marker for notochord and notochord-derived tumors with nearly a 100% expression rate, although it has been reported to be expressed in some germ cell tumors and small cell lung cancer [9, 10].

In this report, we described a patient with a history of familial chordoma who later developed multiple cancers including squamous cell carcinoma (SCC) of the skin, basal cell carcinoma (BCC) of the skin, Hodgkin lymphoma, and aggressive undifferentiated pleomorphic sarcoma (UPS). Genetic mechanisms underlying the pathogenesis of familial chordoma and multiple cancers are discussed.

## Case presentation

**Fig. 1:**
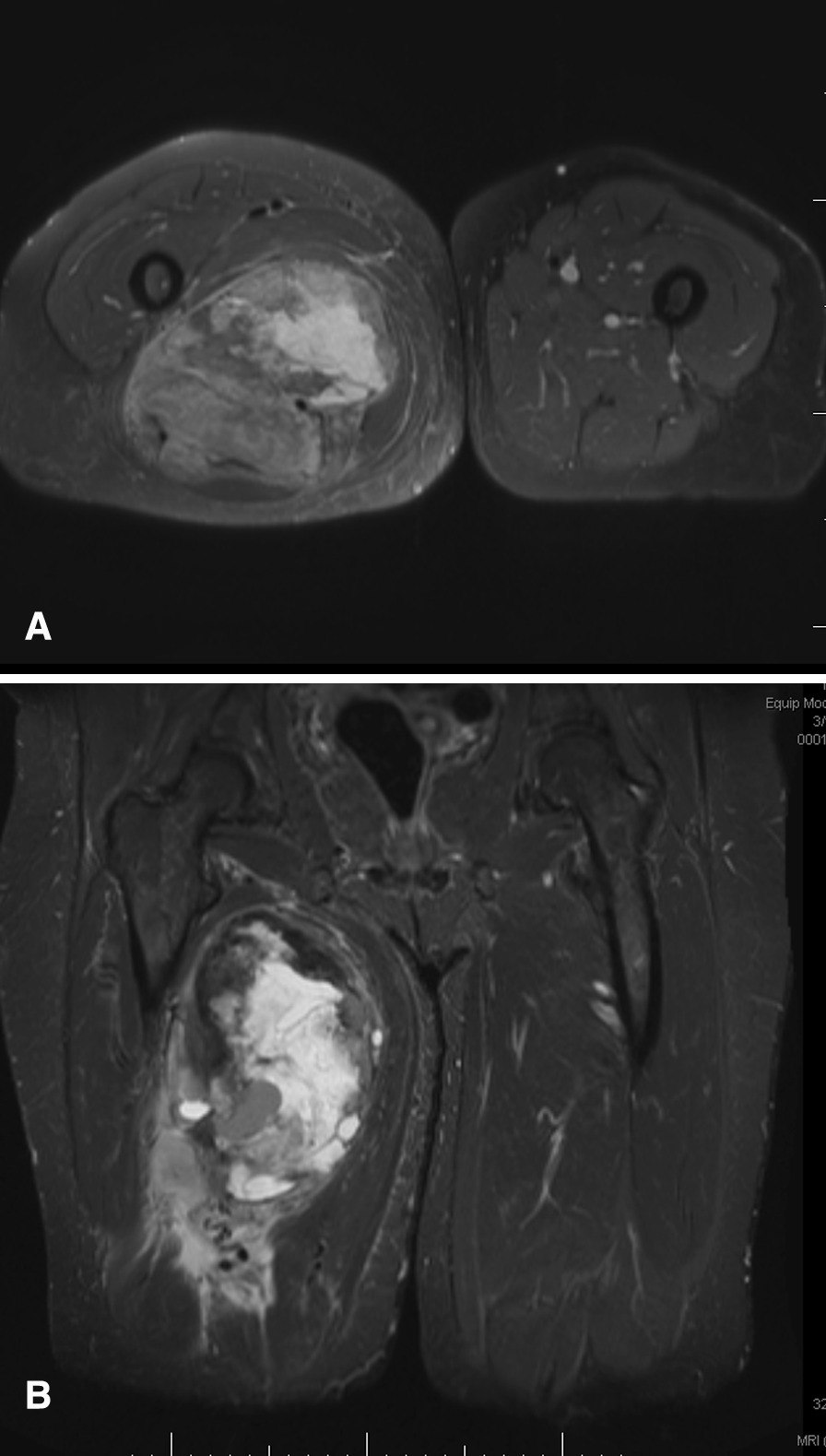
Pre-treatment MRI images. **A** Axial PD fat suppressed MR image of the right thigh demonstrates a heterogeneous soft tissue mass measuring 13.5 × 11.4 cm in transverse and AP dimension; **B** Coronal stir (short T1 inversion recovery) MR image of a heterogenous soft tissue mass measuring 20.8 × 11.3 cm in craniocaudal and transverse measurement

**Fig. 2:**
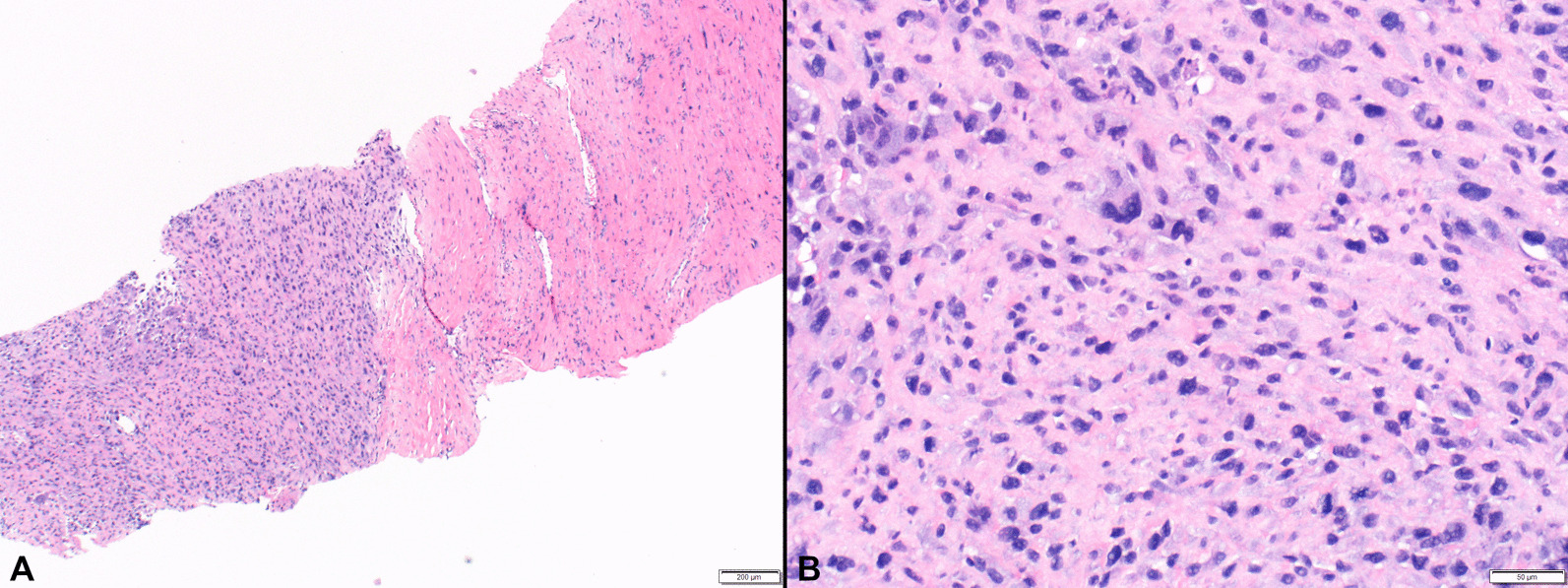
Core needle biopsy specimen showing tumor heterogeneity with high cellularity on the left and stromal collagenization on the right (**A**). Higher magnification image demonstrates a high-grade pleomorphic sarcoma with severe nuclear atypia (**B**). Microscopy was performed using an Olympus BX46 microscope with UPlanFL N lenses and an Olympus DP73 camera with no filter; acquisition software was Olympus cellSens standard with a resolution of 4800 (W) × 3600(H) pixels and no downstream processing

**Fig. 3:**
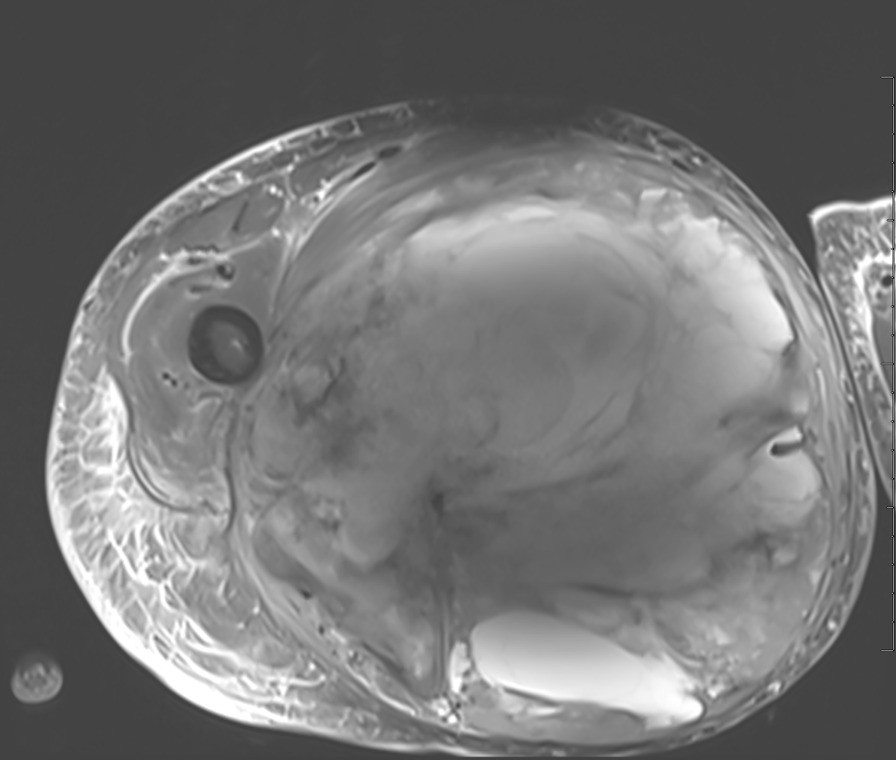
Post-treatment MRI images. Axial PD fat suppressed MR image of the right thigh demonstrates interval increase in the size of the soft tissue mass, measuring 21.6 × 20.9 cm in transverse and AP dimension

**Fig. 4:**
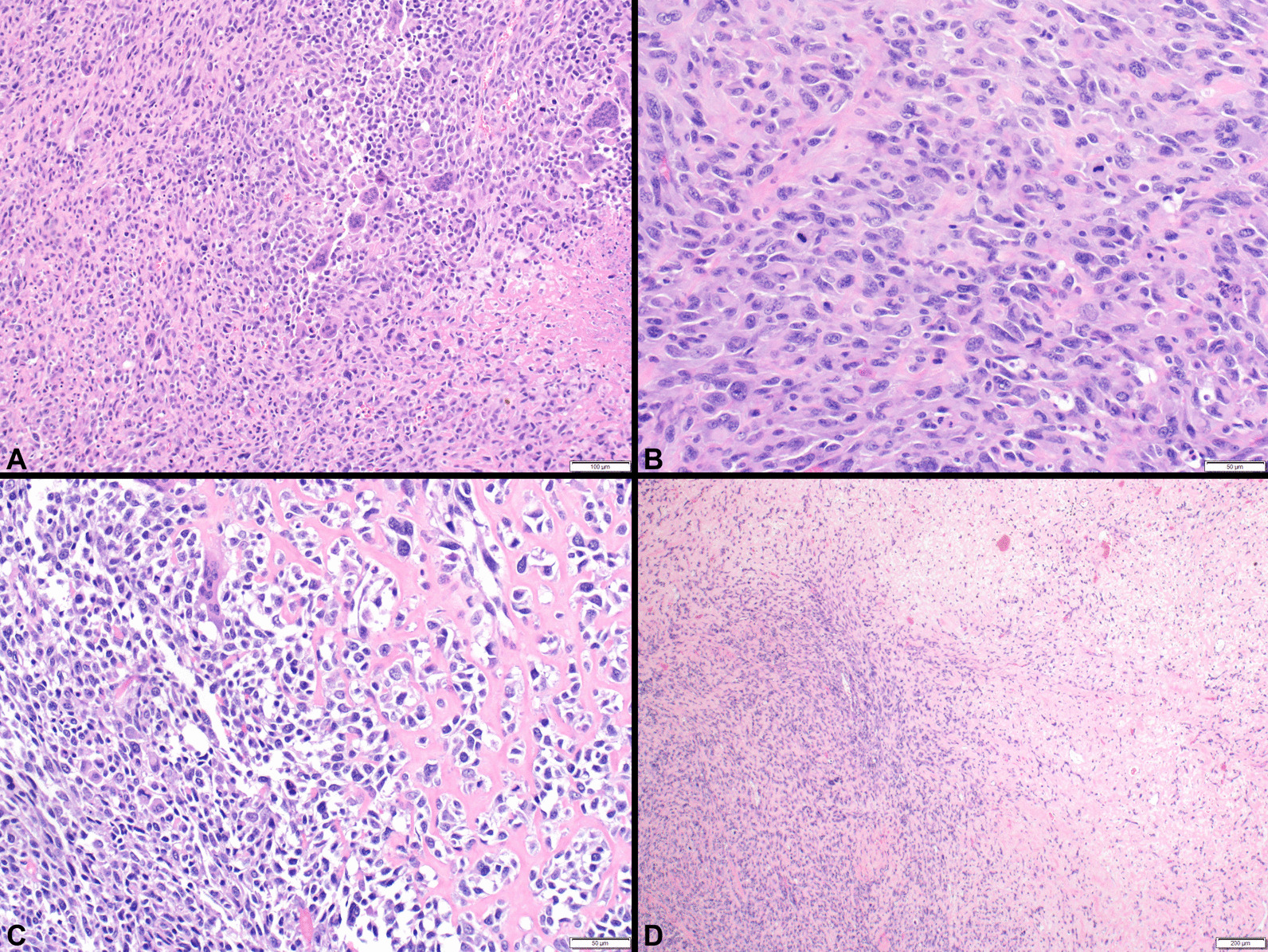
The sarcoma shows very high cellularity with a sheet-like growth pattern, multinucleated giant cells and necrosis (**A**). On higher magnification, there are high-grade pleomorphic tumor cells and multiple mitotic figures (**B**) with areas of heterologous osteosarcomatous differentiation (**C**, right). Chemotherapy effect, characterized by cell death and hyalinization, was present in ~ 60% of the tumor (**D**, right). Microscopy was performed as in Fig. 2

A 67-year-old white woman developed gradually increasing right hip and thigh pain over three months. Physical examination revealed a 20 × 10 cm medial and posterior soft tissue mass in right thigh with moderate tenderness to palpation. Motor power and sensation were intact. An MRI showed an 18.6 × 13 × 11-cm mass within the hamstring musculature (Fig. 1). A biopsy revealed a high-grade UPS (Fig. 2), and a PET-CT revealed bilateral hypermetabolic lung nodules and right inguinal chain hypermetabolic lymphadenopathy consistent with metastatic disease. She was treated with pegylated liposomal doxorubicin (PLD) and infusional ifosfamide with mesna [11]. Repeat imaging demonstrated resolution of the lung nodules after 1 cycle. However, after the 3rd cycle, imaging revealed progression in the primary tumor (21 × 20 × 30-cm) (Fig. 3) and multiple new pulmonary metastases. The primary tumor was surgically excised for symptom control; pathology of the resection specimen revealed a FNCLCC grade 3 UPS with lymphovascular invasion and negative margins with a chemotherapy effect in ~ 60% of the tumor (Fig. 4).

**Fig. 5:**
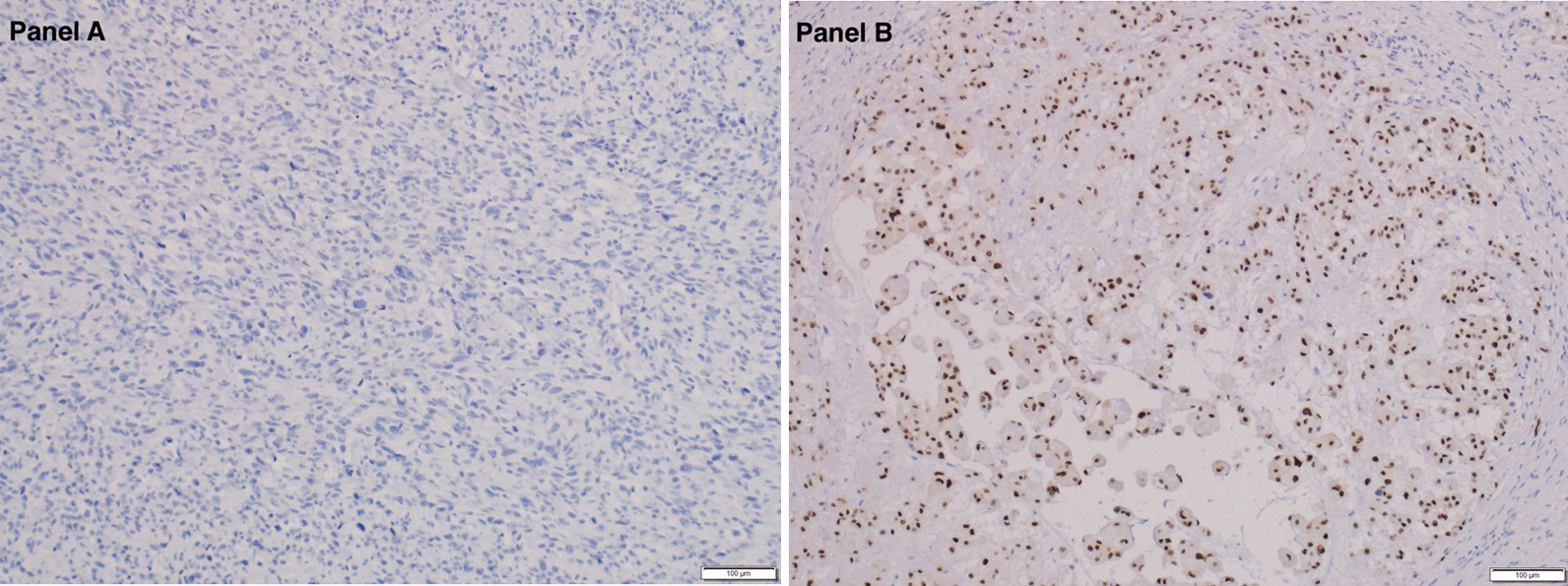
Immunohistochemistry stain for Brachyury is negative in the tumor cells (**A**); a positive control shows brachyury expression in a chordoma (**B**). Microscopy was performed as in Fig. 2

Next generation sequencing (NGS) using the FoundationOne platform of the primary tumor revealed several genetic changes including missense mutation of *AXL* (R368Q) and *RB1* (R661W) and intron 5 rearrangement of *FAS*. The tumor was microsatellite stable and had a tumor mutational burden of 3 mutations/Mb. In addition, several variants of unknown significance were identified, including T535N in *ALK*, R496H in *BRCA1*, L2277F in *BRCA2*, Q740H in *BRIP1*, S301F in *CCT6B*, L219I in *CSF3R*, S1134C in *CUX1*, R127Q in *ETV6*, P197L in *IL7R*, L168* in *JAK3*, K2148N in *MKI67*, I754M in *MSH3*, G1366S in *NOTCH1*, and *ROS1* rearrangement. The *TBXT* gene, which encodes brachyury, is not included in the FoundationOne testing (Additional file 1: Technical specifications of FoundationOne NGS platform. Genes examined and mutations detected). Immunostaining of the primary UPS specimen revealed no detectable nuclear brachyury staining (Fig. 5). Due to disease progression and performance status, treatment with gemcitabine was initiated [12]. The patient only received 1 cycle of treatment before she developed acute hypoxic respiratory failure with bilateral pleural effusions and left pneumothorax, with clinical and imaging findings of disease progression. The treatment regimen was changed to pembrolizumab plus pazopanib [13–15]; although well-tolerated, she developed continued tumor progression and passed away 8 months after diagnosis.

Her past medical history was notable for a spheno-occipital chordoma excised at age 39. Interestingly, ten members of her family were diagnosed with chordoma. Of ten cases, nine involved the clivus or nasopharynx with the age of diagnosis ranging from 6 to 68-years-old. One brother was diagnosed at age 28 with an aggressive sacral chordoma and later died of metastatic disease. The patient and members of her family were involved in extensive genetic studies to identify the genetic abnormality in familial chordoma, which showed *TBXT* gene duplication on 6q27 and contained a SNP variant rs2305089 [5, 6]. Her last follow-up MRI, at age 61, still revealed a stable 1.2 × 0. 8 × 2.3-cm soft tissue mass localized in the posterior nasopharynx appearing to connect a midline defect in the clivus. There was no evidence of progression or metastatic disease, and clinical observation was recommended. There was no history of second malignancy in her other family members with chordoma.

At age 52 she was diagnosed with SCC, which was incidentally found in the pathology specimen after hemorrhoidectomy. This was an invasive carcinoma treated with further surgical excision with no adjuvant therapy. Follow-up high-resolution anoscopy with biopsies revealed no evidence of any residual dysplasia or carcinoma in situ. She also was diagnosed with several superficial BCC lesions, which were removed during the previous 15 years before her death. She also had an adenomatous colonic polyp removed at age 59. At age 62 she developed a gradually enlarging right neck mass over 2 months. A biopsy revealed classical Hodgkin disease, nodular sclerosing type, and PET-CT imaging showed bilateral hypermetabolic supraclavicular, mediastinal, and left hilar adenopathy (stage IIA). She was treated with four cycles of doxorubicin, bleomycin, vinblastine, and dacarbazine as the ABVD regimen; PET-imaging after two cycles revealed a complete response. She completed chemotherapy followed by radiation therapy to the mediastinum, left hilar, and supraclavicular areas. She had no subsequent evidence of lymphoma recurrence. She also had a benign PET-negative thyroid nodule found during work-up for lymphoma that remained stable. She had no significant exposure history, worked in an office, and was a never smoker.

## Discussion and conclusions

We describe a patient with a familial chordoma and a history of multiple cancers throughout her life including Hodgkin disease and UPS. The UPS progressed rapidly on PLD/ifosfamide and subsequent treatments with gemcitabine and then pembrolizumab plus pazopanib. We discuss the biology of chordoma and the use of genetic studies to broaden treatment options for aggressive tumors that do not respond to standard therapy.

Genomic rearrangements, including copy number variants, contribute to disease susceptibility in both sporadic as well as some inherited Mendelian diseases. The patient and members of her family were involved in genetic studies to identify the genetic abnormality underlying the pathogenesis of familial chordoma. High-resolution array-CGH (comparative genomic hybridization) revealed that her rare variant of chordoma is associated with *TBXT* gene duplication on 6q27 and *TBXT* gene sequencing showed an SNP variant rs2305089 (G177D). *TBXT* encodes brachyury, which is important in notochord development and expressed in most sporadic chordomas. All affected individuals in the family shared a common 6q disease-related haplotype. [5, 6]. These findings are in line with data from other studies that previously identified brachyury as a crucial factor in the pathogenesis of chordoma [16–18]. Furthermore, brachyury has been reported to be a driver of cancer stemness and therapy resistance, and may play roles in cancer progression, epithelial-mesenchymal transition (EMT), and metastasis in various types of cancer [19–21] including breast cancer [22–25], prostate cancer [26, 27], non-small cell lung cancer [28–30], colorectal cancer [31], hepatocellular carcinoma [32], and some other epithelial cancers [33–35]. There is no known association between T gene and the other tumors (SCC, BCC, Hodgkin lymphoma, or UPS) that occurred in the patient described here. The mechanism was proposed to be through the Yes-associated protein (YAP) regulatory axis, a key regulator of tissue growth and homeostasis [36]. Amplification of the YAP gene locus has been reported in a wide spectrum of human and murine tumors, and one study found that brachyury enhances YAP transcription by binding with the proximal promoter region to increase its stability post-transcription [37].

UPS is one of the most common subtypes of soft tissue sarcoma. It is characterized by a lack of definite lineage differentiation using currently available diagnostic techniques, and studies suggest several subtypes of soft tissue sarcoma, including liposarcoma and leiomyosarcoma, may evolve into UPS [38]. UPS is a disease with complex genomic alterations, and gene expression studies suggest the existence of functional subgroups of UPS that have different metastatic propensity and clinical outcomes [39–41]. The most commonly identified mutated genes in UPS were *TP53* (66%), *ATRX* genes (34%), and *RB1* (28%); although the frequency varies from study to study, there are consistent trends among these 3 genes [42–44]. Nonetheless, there are very few clinically targetable mutations identified to date [45].

**Fig. 6:**
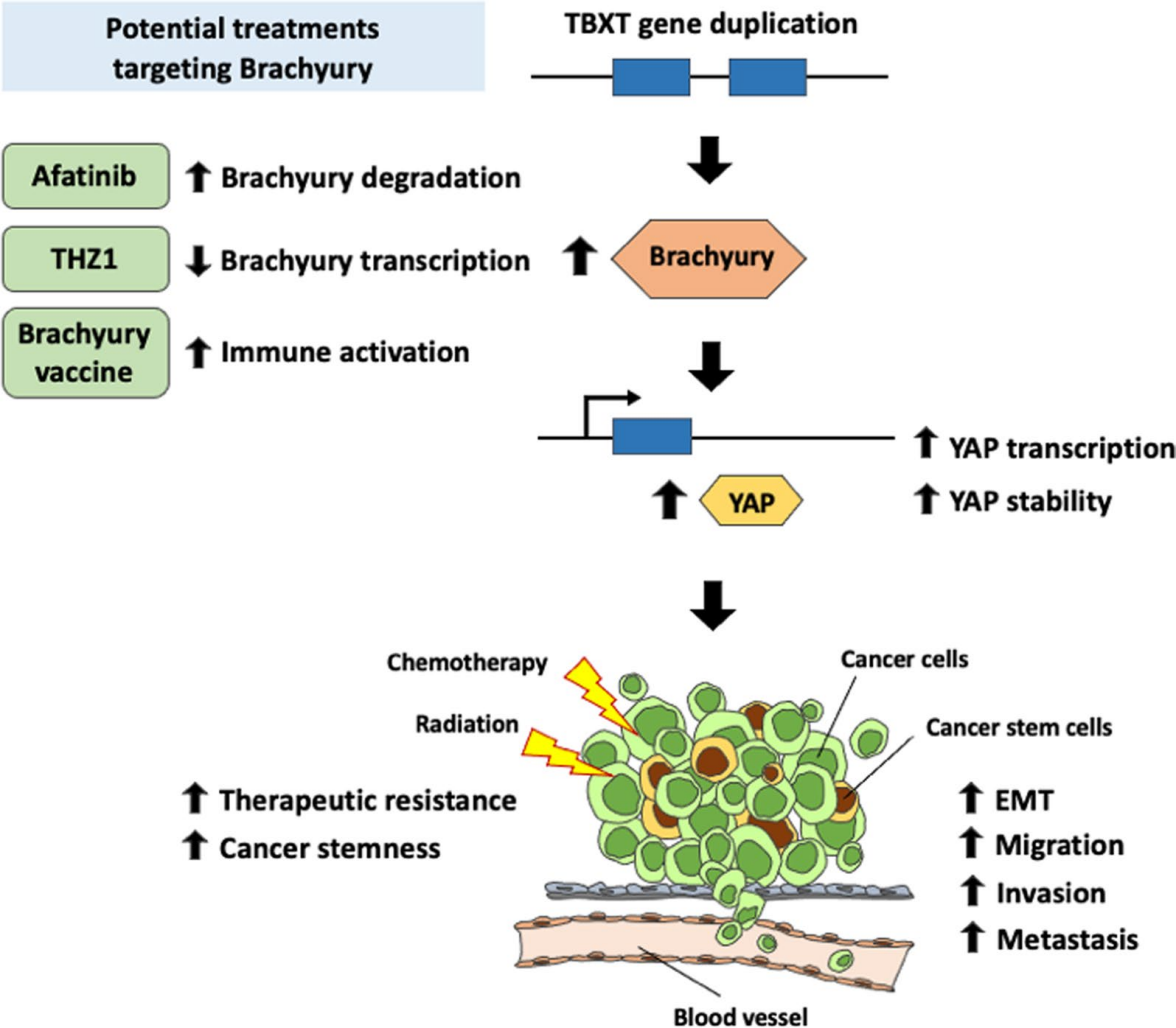
Potential effects of brachyury in promoting cancer aggressiveness. Over-expression of brachyury promotes cancer stemness and resistance to chemotherapy and radiation, and also enhances epithelial-mesenchymal transition, migration, invasion, and, eventually, metastasis. The mechanism is not well defined, but may relate to up-regulation of YAP transcription and stabilization of YAP protein by increased production of brachyury, with subsequent effects mediated primarily by YAP. This is an original figure

Given her known status of *TBXT* gene duplication, brachyury might play a role in both the development and the aggressiveness and resistance to therapy of the UPS in her case (Fig. 6). Therapy that has demonstrated efficacy in pre-clinical studies of chordoma include afatinib, an epidermal growth factor receptor (EGFR) inhibitor, and THZ1, a cyclin-dependent kinase (CDK) 7/12/13 inhibitor [46, 47]. Afatinib was the only EGFR inhibitor that inhibited the proliferation of all chordoma cell lines tested, and its antiproliferative activity correlated with the ability to promote degradation of EGFR and brachyury [46]. CDK inhibitors targeting CDK7/12/13 and CDK9 have also been found to suppress chordoma cell proliferation, reduce tumor growth in vivo, and decrease brachyury protein expression in these systems [47]. The transcription-associated CDKs, including CDK7, CDK8, CDK9, CDK12 and CDK13, are important regulators of gene expression [48], and transcription-associated CDK inhibitors have been found to down-regulate highly expressed, enhancer-associated transcription factors in other cancers [47]. *TBXT* is associated with a 1.5 Mb region containing strong enhancers or “super-enhancers,” and is the most highly expressed super-enhancer associated transcription factor in chordomas [47]. Therefore, transcription-associated CDK inhibitors may exert their action by down-regulating brachyury [47, 49], providing an example of transcription factor down-regulation by a small molecule. Various strategies to target brachyury are currently under investigation in clinical trials (Table 1). Results of early phase trials of a brachyury vaccine demonstrated induction of an immune responses to brachyury and showed some evidence of clinical benefit in patients with chordoma and metastatic solid tumors [50–52].

**Table 1:** Summary of therapy targeting brachyury protein in cancer

Treatment	Mechanism	Disease model	Stage	Result	Ref
Yeast-Based vaccine	Immune stimulation	metastatic or unresectable locally advanced malignant solid tumors	Clinical phase I	Induce T-cell response. No serious adverse effects. PR 10%; SD 80%; PD 10%. Currently ongoing phase II study (NCT02383498)	[50]
DNA Nanoparticle-Mediated shRNA	shRNA inhibit brachyury expression	Chordoma	Pre-clinical	Induced apoptosis, upregulated the epithelial biomarker, E-cadherin, downregulated the mesenchymal biomarker, Snail and Slug, and suppressed cell growth	[53]
Poxviral TRICOM-Based Vaccine	Immune stimulation	metastatic or unresectable locally advanced malignant solid tumors	Clinical phase I	Induce T-cell response. No serious adverse effects. SD 45%; PD 55%	[51]
Afatinib	Brachyury degradation	Chordoma	Pre-clinical	Antitumor efficacy in U-CH1, SF8894, CF322, and CF365 chordoma tumor models in vivo. Currently ongoing phase II study in EGFR expressing chordoma (NCT03083678) and metastatic or unresectable chordoma (EUDRACT 2016-002766-31)	[46]
THZ1 (CDK 7/12/13 inhibitors)	Down-regulation of brachyury expression	Chordoma	Pre-clinical	Can reduce tumor growth in vivo	[47]
Modified Vaccinia Ankara Priming Vaccine	Immune stimulation	metastatic or unresectable locally advanced malignant solid tumors	Clinical phase I	Well tolerated and induces immune responses to brachyury. SD 60%; PD 40%	[52]
H3K27 demethylase inhibitors	Epigenetic silencing of TBXT	Chordoma	Pre-clinical	Pharmacologic inhibition of H3K27-demethylases promotes chordoma cell death	[54]

In a panel of soft tissue sarcomas, 0/60 UPS cases not associated with chordoma were found to have nuclear expression of brachyury. Interestingly, in one study, 75/76 chordomas had nuclear brachyury expression, while the one negative case exhibited sarcomatous transformation. Thus, the finding that our UPS case did not express brachyury does not exclude that it could have arisen from a chordoma, although the location of the tumor would be very atypical [10]. On a tissue-based NGS study (FoundationOne®), the UPS tumor of the patient described here demonstrated microsatellite stability, low tumor mutational burden, and mutations in *AXL* and *RB1*. AXL activation could predict resistance to EGFR inhibitors [55, 56]. However, no known clinical significance on the effect of the *AXL* missense mutation in this case is known. *RB1* inactivation, predicted by a missense mutation in the pocket domain (aa 773–928) as seen in this case, may be associated with sensitivity of Aurora kinase A and resistance to CDK4/6 inhibitors, but this is also not clinically targetable at present [57]. *ROS1* rearrangement, also found in our patient’s tumor, is a common event in carcinogenesis and has been demonstrated in a variety of human cancers, including glioblastoma, non-small cell lung cancer (NSCLC), and sarcomas, such as angiosarcoma and epithelioid hemangioendothelioma [58]. Thus, her tumor may have been responsive to *ROS1* tyrosine kinase inhibitors, such as crizotinib, although she did not receive that trial agent; most of the studies to date have focused on lung cancer models [59, 60]. Interestingly, despite being the most common gene with genetic alteration in UPS, there was no *TP53* mutation found on the FoundationOne platform in this patient (Additional file 1). Several variants of unknown significance were also detected as described above, but their association with malignancy is currently unknown.

In this patient with multiple rare tumors including a UPS with an aggressive nature, multiple genetic alterations such as *AXL* and *RB1* mutation might play a role. However, the occurrence of multiple uncommon tumors suggests an underlying susceptibility, and the presence of the germline *TBXT* duplication may have an important role in the pathogenesis of her tumors and their biology. Detailed molecular and genetic studies could offer therapeutic targets to alleviate the progression of disease in the future.

## Supplementary Information

**Additional file 1**. Technical specifications of FoundationOne NGS platform. Genes examined and mutation detected.

## Data Availability

Not applicable as all data is shown in Additional file 1.

## Abbreviations

BCC: Basal cell carcinoma
CDK: Cyclin-dependent kinase
EGFR: Epidermal growth factor receptor
EMT: Epithelial-mesenchymal transition
FNCLCC: French Federation of Cancer Centers Sarcoma Group
MRI: Magnetic resonance imaging
NGS: Next-generation sequencing
NSCLC: Non-small cell lung cancer
PET-CT: Positron emission tomography-computed tomography
PLD: Pegylated liposomal doxorubicin
SCC: Squamous cell carcinoma
SNP: Single-nucleotide polymorphism
TBXT: T-box transcription factor T
UPS: Undifferentiated pleomorphic sarcoma
YAP: Yes-associated protein
