# Supplementary material for: Multiple malignant tumors in a patient with familial chordoma, a case report
Source: BMC Med Genomics. 2021 Aug 31;14:213. doi: 10.1186/s12920-021-01064-0 (PMC8406958; doi:10.1186/s12920-021-01064-0)
Supplement: Supplementary file 1 — Additional file 1. Technical specifications of FoundationOne NGS platform. Genes examined and mutation detected. [file 12920_2021_1064_MOESM1_ESM.docx]

**Supplementary data**

FoundationOne^®^ technical specifications:

<https://assets.ctfassets.net/w98cd481qyp0/42r1cTE8VR4137CaHrsaen/baf91080cb3d78a52ada10c6358fa130/FoundationOne_Heme_Technical_Specifications.pdf>

Genomic alterations reported:

R368Q in *AXL,* R661W in *RB1,* intron 5 rearrangement in *FAS*, T535N in *ALK*, R496H in *BRCA1*, L2277F in *BRCA2*, Q740H in *BRIP1*, S301F in *CCT6B*, L219I in *CSF3R*, S1134C in *CUX1*, R127Q in *ETV6*, P197L in *IL7R*, L168* in *JAK3*, K2148N in *MKI67*, I754M in *MSH3*, G1366S in *NOTCH1*, and *ROS1* rearrangement.
